# Psilocybin in the treatment of eating disorders: a systematic review of the literature and registered clinical trials

**DOI:** 10.1007/s40519-025-01771-y

**Published:** 2025-07-29

**Authors:** Francesco Bevione, Maria Carla Lacidogna, Raffaele Lavalle, Giovanni Abbate Daga, Antonio Preti

**Affiliations:** Department of Neuroscience, University of Turin, Via Cherasco 15, 10126 Turin, Italy

**Keywords:** Eating disorders, Psilocybin, Psychedelics, Psychotherapy, Novel treatments

## Abstract

**Background:**

Fluoxetine remains the only pharmacological treatment approved for Bulimia Nervosa, and no other drugs have been approved for eating disorders (EDs). The rationale for exploring psilocybin as a treatment for EDs is compelling, both from biological and psychological perspectives. Moreover, its safety profile in healthy individuals appears favorable. This systematic review aims to examine original research articles and registered clinical trials to assess the current psilocybin’s therapeutic potential in EDs.

**Methods:**

Systematic review following the indications of the Preferred Reporting Items for Systematic Reviews and Meta-Analyses (PRISMA) guidelines. We searched PubMed, Excerpta Medica Database (EMBASE), and the Cochrane Library from inception until 29 July 2024, with key terms: “psilocybin” and “eating disorders”. Quality was assessed through the *Quality Assessment Tool for Before–After (Pre–Post) Studies With No Control Group* released by the National Heart, Lung, and Blood Institute (NHLBI). We performed an additional search on the registry of clinical trials available at the website https://clinicaltrials.gov.

**Results:**

Two studies met the inclusion criteria for our analysis. The first was an open-label feasibility study involving 10 individuals with Anorexia Nervosa (AN), without a control group. The second was a single case report describing the use of psilocybin in a person with AN. In addition, six registered clinical trials of psilocybin in individuals with EDs were identified.

**Conclusions:**

The initial evidence shows that psilocybin might be safe and well-tolerated in AN. The promising results and the need for tests in enlarged samples encourage further research on psilocybin in EDs.

**Level of evidence VIII:**

Evidence from nonrandomized controlled clinical trials, nonrandomized clinical trials, cohort studies, case series, case reports, and individual qualitative studies.

**Supplementary Information:**

The online version contains supplementary material available at 10.1007/s40519-025-01771-y.

## Introduction

Feeding and eating disorders are mental disorders characterized by persistently disturbed eating or eating-related behavior causing altered consumption or absorption of food, and significantly impairing physical health and psychosocial functioning [1]. The main eating disorders in the current classification are Anorexia Nervosa (AN), Bulimia Nervosa (BN), and Binge Eating Disorder (BED) [1, 2].

AN, BN, and BED all can evolve with a chronic course and suffer from enhanced mortality from medical complications and suicide [3–9]. Current interventions for AN primarily focus on nutritional rehabilitation, medical monitoring, and the management of psychiatric comorbidities [3, 10–12]. For BN and BED, psychotherapeutic and pharmacological approaches have shown some efficacy, although their long-term effectiveness remains uncertain [2, 13].

Unfortunately, more than 20% of persons who suffer from AN, and up to 40% according to some studies [5, 13], do not respond to treatments, and the course of the disease lasts lifelong [14]. Alarmingly, these data have not improved during the last decades [5]. Some authors refer to cases of AN lasting more than 7 years, with no treatment response and persistent extremely low weight, as *'Severe Enduring Anorexia Nervosa*' (SE-AN) [15].

As for BN and BED, information on resistance to treatment is limited, and a chronic course is reported in about 20% of cases [16, 17]. In addition, both in BN and BED relapse is frequent after a phase of stabilization [5].

Due to the scarcity of effective treatments, new therapeutic options are being tested, including novel psychotherapy techniques, neuromodulation, and virtual reality [12, 18, 19]. While initial results are promising, these interventions are still in the experimental stage and not yet ready for clinical practice.

In the wake of the findings obtained in other diseases, such as depression, obsessive–compulsive disorder (OCD), and substance use disorder (SUD), psilocybin has been proposed among the new treatments that could restore hope to people who suffer from SE-AN [20–24]. Psilocybin belongs to the pharmaceutical class of psychedelics. These molecules are thought to mediate their neurobiological mechanisms through the effects on the serotonergic, via 5-HT_2A_ and 5-HT_1A_ receptors, and glutamatergic systems, via N-methyl-d-aspartate (NMDA) and α-amino-3-hydroxy-5-methyl-4-isoxazolepropionic acid (AMPA) receptors [25]. The main actions of psychedelics seem to be the increase of neuroplasticity, immunomodulation, and modulation of serotonergic, dopaminergic, glutamatergic, GABAergic, and norepinephrinergic receptors, transporters, and turnover systems, as highlighted by preclinical and clinical studies [25, 26].

The rationale for utilizing psilocybin in AN is particularly intriguing. From a biological point of view, psilocybin is an agonist of 5-HT_2a_ serotonin receptors and increases the concentrations of BDNF, both significantly impaired in persons with active AN, and promotes gray matter restoration [27–30]. Furthermore, the administration of psilocybin helps restart cortical neuroplasticity, especially in areas responsible for emotional regulation [31, 32]. In neuroimaging studies, psilocybin was shown to decrease the activity and functionality of the *default mode network* (DMN) and the anterior cingulate cortex (CCA) while increasing the insula activity [30, 33–35]. These areas are, respectively, hyper- and hypo-active in persons who suffer from AN, and these alterations are believed to be responsible for cognitive rigidity and the tendency to ruminate [36, 37]. Studies investigating the neurobiological effects of psychedelics in animal models, including the activity-based anorexia (ABA) rodent model, have indeed confirmed the benefits of the administration of psilocybin on activity-based anorexia in female rats by enhancing cognitive flexibility [38, 39]. It has been proposed that the therapeutical actions of psilocybin might be at least partially mediated by the activation of serotonin receptors in the gut via the direct stimulation of the vagus nerve [40]. In this context, psilocybin might also interact with the microbiota–gut–brain axis [40, 41].

The most promising potential of psilocybin is from a psychological point of view. Authors suggest that *psychedelic-assisted therapy* (PAT) could help persons who suffer from AN leave negative beliefs regarding body image, regulate the reward system, reduce pathological avoidance of social situations, and surpass traumatic experiences [32, 33, 42–48]. Finally, the promising results of psilocybin in treating other diseases allow us to suppose a potential benefit in the cure of the psychiatric comorbidities of AN [20–24, 49].

The safety profile of psilocybin is considerably favorable under monitoring, and toxic effects are rare even in non-controlled contexts. In addition, there have never been reported cases of addiction or psychotic onset [24, 30, 33]. The comparison of potential benefits and possible adverse effects is so convenient that many authors expressed the urge for funding in this field to reach commercialization as soon as possible [50–53]. Regardless, it is reasonable to worry that psilocybin effects in persons suffering from AN could be different from those in healthy people. Studies of tolerance and acceptability are required to solve such doubts before psilocybin can be allowed for therapeutic use in AN.

In BN, case reports employing ketamine, a dissociative anesthetic with hallucinogenic effects, showed promising results on remission from binge-purging behavior and mood intolerance [54–56]. Ayahuasca also produced self-reported improvements in disordered eating behaviors in persons suffering from BN [57, 58]. Psychedelics have not been employed in the treatment of BED yet. However, the benefits of the reward system [42, 44], emotion regulation [31], trauma processing [47], and depression [23, 24] open the rationale for a favorable utilization in BED also.

### Aims of this systematic review

Treatment options for EDs are limited, and the rationale for the employment of psilocybin is solid. We aimed to conduct a systematic review of the literature to assess whether the potential of psilocybin in EDs is currently under proper investigation. In addition, if preliminary results are available, we aimed to evaluate whether the findings promote, or discourage, further experimentation on this molecule.

The PICO framework was as simple as possible. The considered population was the population of people suffering from EDs as diagnosed by clinicians; the intervention was the utilization of psilocybin in any type of study; the control was defined according to the study parameters; since psilocybin is a novel therapeutic option, we acknowledged that a control group might not be provided; the outcome was any outcome provided by the included studies.

## Methods

The reporting of this systematic review follows the indications of the Preferred Reporting Items for Systematic Reviews and Meta-Analyses (PRISMA) guidelines [59, 60]. The protocol for this systematic review was registered with PROSPERO (CRD42024580338).

### Inclusion and exclusion criteria

Studies were included when they were original articles reporting data on the utilization of psilocybin in people suffering from EDs. To provide an analysis as more exhaustive, we included any type of study.

### Search methods

We searched PubMed, Excerpta Medica Database (EMBASE), and the Cochrane Library without time limitations from inception until 29 July 2024. The following key terms were used: ("psilocybin"[MeSH Terms] OR "psilocybin"[All Fields] OR "psilocybin"[All Fields] OR "psilocybin s"[All Fields]) AND ("feeding and eating disorders"[MeSH Terms] OR ("feeding"[All Fields] AND "eating"[All Fields] AND "disorders"[All Fields]) OR "feeding and eating disorders"[All Fields] OR ("eating"[All Fields] AND "disorders"[All Fields]) OR "eating disorders"[All Fields]). The search was limited to original articles. No gender, age, ethnicity, or language restriction was applied.

On 12 August 2024, we searched the registry of clinical trials available at the website https://clinicaltrials.gov. The “condition/disease” term was “eating disorders”; the “intervention/treatment” term was “psilocybin”. We included any registered protocol of study on the employment of psilocybin in people suffering from EDs.

### Studies’ selection

**Fig. 1:**
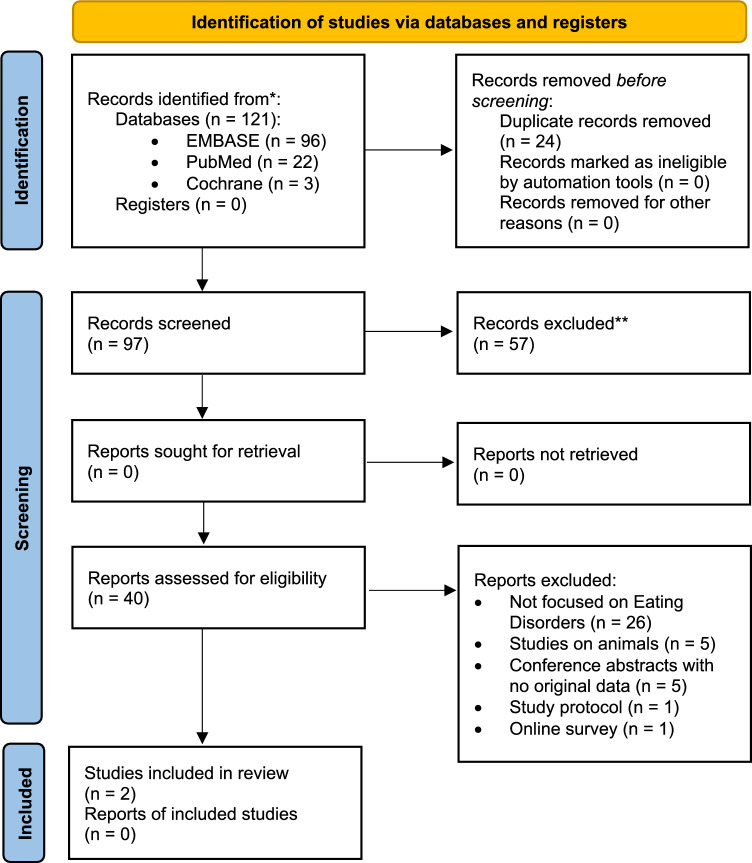
PRISMA 2020 flow diagram for the studies on psilocybin in eating disorders

Three authors (FB, MCL, AP) examined the list of articles and decided whether the title and abstract were compatible with the inclusion criteria. Discrepancies were solved by a discussion with a fourth experienced researcher (GAD). Selected results were then meticulously re-evaluated in their full text to confirm that they fulfilled the inclusion criteria. In addition, their references section was inspected to retrieve any missed articles (Fig. 1).

Every registered trial on the website https://clinicaltrials.gov was individually assessed to verify it concerned the employment of psilocybin in EDs.

### Data extraction

Three evaluators (FB, MCL, AP) extracted the following data from each article: author(s) and year of publication; type of study; sample size (n); mean age and gender; specific ED diagnosis; baseline BMI; years of illness; treatment; primary outcomes; secondary outcomes; adverse effects; and main findings. Discrepancies in data extraction were solved by a discussion with a fourth experienced researcher (GAD).

The same researchers extracted the following data from each clinical trial: name of the clinical trial; ID; date of submission, date of start, date of completion; type of study; clinical phase; sample size; status of the study; location; specific ED diagnosis; age of inclusion; treatment; design of the study; outcomes; tools to assess outcomes; endpoints; whether the results have been posted.

### Data synthesis

Data were tabulated to provide a better global view of information.

In Table 1, we reported any information regarding the published articles included.

**Table 1:** Characteristics and main findings of the included studies

Authors and year	Type of study	n	Mean age (SD)	Gender	Diagnosis	Baseline BMI (SD)	Years of illness (SD)	Treatment	Primary outcomes	Secondary outcomes	Adverse effects	Main findings
Verroust et al., 2021	Case study	1	35.0	100% female	AN-BP current	14.0	16.0	2 injections of psilocybin of unknown dose at a distance of 4 days	Weight gain	Emotions diary after injections	NR	7 kg increase after 1 month (BMI from 14.0 to 16.4)
Peck et al., 2023	Open-label feasibility study	10	28.3 (3.7)	100% female	AN-R current (40%) AN-BP current (10%) AN-R partial remission (50%)	19.7 (3.7)	8.9 (5.9)	Single 25 mg dose of psilocybin in conjunction with psychological support	Safety profile: Vital signs ECG Clinical laboratory tests Suicidality assessed with C-SSRS	EDE-Q BMI PASTAS STAI YBC–EDS Qualitative perception of the treatment QIDS CIA RMQ	Headache (80%) Fatigue (70%) Nausea (30%) Feeling abnormal (20%) Migraine (20%) Dizziness (20%) Illusion (20%) Pain (10%) Anxiety (10%) Heart rate response increased (10%) Abdominal pain (10%) Hypoglycemia (20%)	Adverse effects were mild and transient One case of hypoglycemia which resolved within 24 h; no other laboratory alterations No increase in suicidal ideation; no suicidal behaviors in the post-dosing follow-up visits Weight concerns significantly decreased at 1-monh and 3-month follow-up with a medium to large effect Shape concerns decreased at 1 month but no longer significant at 3-month follow-up Eating concerns significantly decreased at 3-month follow-up Changes on dietary restraint were not significant Changes in BMI were not significant At 1-month follow-up significantly decreased the PASTAS, STAI-T, and YBC–EDS scores CIA scores decreased at 1-month follow-up Overall, the experience was regarded as meaningful and well-accepted by all participants

n: numerousness; SD: Standard Deviation; BMI: Body Mass Index; AN-BP: Anorexia Nervosa Binge-Purging subtype; NR: Not Reported; AN-R: Anorexia Nervosa Restricting subtype; ECG: Electrocardiogram; C-SSRS: Columbia-Suicide Severity Rating Scale; EDE-Q: Eating Disorder Examination Questionnaire; STAI: State–Trait Anxiety Inventory; PASTAS: Physical Appearance State and Trait Anxiety Scale; YBC–EDS: Yale–Brown–Cornell–Eating Disorders Scale; QIDS: Quick Inventory of Depressive Symptomatology; CIA: Clinical Impairment Assessment; RMQ: Readiness and Motivation Questionnaire

In Table 2, we described the characteristics of the currently ongoing trials.

**Table 2:** Characteristics of the published trials with psilocybin in eating disorders

Clinical trial	ID	Dates	Type of study	Phase	N	Status	Location	Condition	Age	Treatment	Design	Outcomes	Tools to assess outcomes	Endpoints	Results posted
Evaluation of Psilocybin (TRP-8802) in the Treatment of Binge Eating Disorder	NCT05035927	2021-08-24 (submission) 2022-03-16 (start, actual) 2025-08 (completion, estimated)	Open label	Phase 2	10 (estimated)	Active, not recruiting	Gainesville, Florida, United States	Binge Eating Disorder	18–64 years	A single 25 mg oral dose of psilocybin will be administered following 6 to 8 h of preparatory psychotherapy. Subjects will be in the study for 12 weeks following the dose of psilocybin	Single-group assignment	Safety Feasibility Magnitude and duration of psilocybin dissociative effects Frequency of binge eating episodes Waist circumference Weight	Adverse effects monitoring Laboratory tests Vital signs MEQ30 MRS Frequency of binge eating episodes Waist circumference BMI	6 weeks 12 weeks	No
Evaluation of Psilocybin in Anorexia Nervosa: Safety and Efficacy	NCT04661514	2020-11-19 (submission) 2021-05-01 (start, actual) 2022-06-10 (completion, actual)	Open label	Phase 2	16 (actual)	Completed	La Jolla, California, United States	Anorexia Nervosa	18–40 years	Psilocybin-assisted psychotherapy: each participant will receive 1 × 25 mg treatment bottle containing 5 × 5 mg oral capsules of psilocybin. The administration session will last approximately 4–6 h and will be supported by a lead therapist and an assisting therapist	Single-group assignment	Safety Feasibility Suicidality Eating psychopathology Weight General psychopathology Consciousness alteration	Adverse effects monitoring ECG Laboratory tests Vital signs Questionnaire of acceptability C-SSRS EDE-Q BMI STAI PASTAS BISSYBC–EDS–SRQ EDI EDE–QS QIDS CIA VAS for hunger, fullness and desire to eat ED-RR 5D-ASC	Baseline Day 1 Day 7 Day 28	Yes
Efficacy and Safety of COMP360 Psilocybin Therapy in Anorexia Nervosa: a Proof-of-concept Study	NCT05481736	2022-07-28 (submission) 2022-10-12 (start, actual) 2024–06 (completion, estimated)	RCT	Phase 2	60 (estimated)	Recruiting	San Diego, California, United States Baltimore, Maryland, United States New York, New York, United States Austin, Texas, United States Dublin, Ireland London, United Kingdom	Anorexia Nervosa	18 years or above	Experimental: 25 mg psilocybin Active Comparator: 1 mg psilocybin	Parallel assignment Randomized allocation Quadruple masking	Safety Eating psychopathology General psychopathology Weight	Adverse events EDE Y-BOCS BMI	4 weeks 12 weeks	No
Effects of Psilocybin in Anorexia Nervosa	NCT04052568	2019-08-08 (submission) 2019-08-26 (start, actual) 2023-04-20 (completion, actual)	Open label	Phase 1	22 (actual)	Completed	Baltimore, Maryland, United States	Anorexia Nervosa—restricting subtype	18–65 years	Participants will undergo up to four psilocybin sessions. Dosing at the first session will be 20 mg. For subsequent sessions participants will either remain at their previous dose, or increase by increments of 5 mg up to a maximum of 30 mg	Single-group assignment	Eating psychopathology General psychopathology Weight Health-related quality of life	HADS EDQLS EDE-Q EDE BMI ANSOCQ	1 week 1 month 2 months 3 months	No
Psilocybin as a Treatment for Anorexia Nervosa: A Pilot Study	NCT04505189	2020-07-30 2021-05-28 (start, actual) 2024-06-12 (completion, actual)	Open label	Phase 1 Phase 2	21 (actual)	Completed	London, United Kingdom	Anorexia Nervosa	21–65 years	Psilocybin-assisted psychotherapy: all participants will receive 3 doses of psilocybin; the maximum dose a participant will receive in a single session is 25 mg	Single-group assignment	Eating psychopathology fMRI	EDE EDE-Q RMQ fMRI	6 months	No
Study of Psilocybin for Anorexia in Young Adults	NCT06399263	2024-04-30 (submission) 2024–06 (start, estimated) 2029–06 (completion, estimated)	Open label	Phase 2	40 (estimated)	Not yet recruiting	San Francisco, California, United States	Anorexia Nervosa	18–25 years	The psilocybin therapy will include three preparatory sessions, psilocybin dosing session one (20 mg), two integration sessions, psilocybin dosing session two (30 mg), and four final integration sessions	Single-group assignment	Eating psychopathology	EDE	Baseline 28 days 90 days 365 days	No

N: numerousness; MEQ30: Mystical Experience Questionnaire; MRS: Magnetic Resonance Spectroscopy; BMI: Body Mass Index; C-SSRS: Columbia-Suicide Severity Rating Scale; EDE-Q: Eating Disorder Examination Questionnaire; STAI: State–Trait Anxiety Inventory; PASTAS: Physical Appearance State and Trait Anxiety Scale; BISS: Body Image States Scale; YBC–EDS–SRQ: Yale-Brown-Cornell–Eating Disorders Scale–Self-Report Questionnaire; EDI: Eating Disorder Inventory; EDE-QS: Eating Disorder Examination Questionnaire-Short; QIDS: Quick Inventory of Depressive Symptomatology; CIA: Clinical Impairment Assessment; VAS: Visual Analog Scale; ED-RR: Eating Disorders Recovery Questionnaire; 5D-ASC: Five Dimensional Altered States of Consciousness; EDE: Eating Disorder Examination; Y-BOCS: Yale-Brown Obsessive Compulsive Scale; HADS: Hospital Anxiety and Depression Scale; EDQLS: Eating Disorder Quality of Life Scale; ANSOCQ: Anorexia Nervosa Stages of Change Questionnaire; RMQ: Readiness and Motivation Questionnaire; fMRI: Functional Magnetic Resonance Imaging

## Results

### Original articles

Overall, 121 results were found with the key terms utilized. Of them, 24 were discarded as duplicates. The other 97 results were inspected in their title and abstract, with a quick screen of the text, to assess whether they fulfilled the inclusion criteria. Indeed, 57 were excluded; 38 were reviews, 8 were notes, 4 were editorials, 3 were letters, 2 were chapters of books, and 2 were out of topic. The remaining 40 articles were meticulously assessed in their full text and supplementary material, and their references were inspected to retrieve any missed results. 38 were excluded; 26 were not focused on EDs, 5 were studies on animals, 5 were conference abstracts not providing any original data, 1 was a protocol of study, and 1 was an online survey evaluating people who use auto-prescribed microdoses of psilocybin. Ultimately, 2 articles were included in our analysis.

The characteristics and main findings of the included studies are summarized in Table 1.

One [61] was the translation in English of a case study conducted in France in 1959 [62]. The article reported the clinical case of a 35-year-old woman suffering from AN-BP who received two injections of unreported doses of psilocybin at a distance of 4 days. The patient was described as a perfectionist, obsessive and self-centered, whose emotional and sexual life was null, and of a great religiousness. The illness started 16 years earlier, and at the moment of the study her weight was 40 kg for 1.69 m (BMI = 14.0 kg/m^2^). The patient also presented depressive-like symptoms, including asthenia, insomnia, negative thoughts, and lack of confidence. At the entry to the hospitalization, the patient received a treatment of Chlorpromazine 100 mg/die, but it produced no benefits on mood or weight after 3 weeks. At that point, clinicians suspended Chlorpromazine and prescribed the first, and after 4 days the second, dose of psilocybin. The two injections produced a semi-confusional state with physical discomfort and dream-like scenes of mystical themes for around 30 min, and later a phase of emotional relief that lasted close to 2 h, during which the patient was able to express about her problems with body, food, sexual difficulties, depression, and she explained about emotional frustration, relationship with her parents, feelings of guilt, and feelings of inferiority. The clinicians reported the recovery as immediate. The day following the second injection, the participant was euphoric. The weight increased remarkably. At the moment of discharge, 1 month later, it was 7 kg higher (from 40 to 47 kg; BMI from 14.0 to 16.4). The patient carried on the follow-up. No adverse effects were reported.

The other was an open-label feasibility study on the safety, tolerability, and exploratory efficacy of psilocybin conducted among people who suffer from AN [63]. Ten females with AN were recruited. Nine were diagnosed with AN-R (4 active, 5 in partial remission), and 1 with AN-BP. The sample was characterized by a long duration of the disease (mean years of illness = 8.9). The treatment consisted of a dose of 25 mg of synthetic psilocybin in conjunction with psychological support. Two psychologists provided support and assessed for safety for 8 h after the psilocybin was administered.

The primary outcomes of the study were safety and tolerability. To evaluate them, vital signs, electrocardiogram (ECG), clinical laboratory tests, and suicidality (with the Columbia-Suicide Severity Rating Scale, C-SSRS) were assessed at baseline, 1 day after, and 7 days after having received treatment. Vital signs and ECG did not reveal any significant changes. The only laboratory test alterations consisted of two cases of hypoglycemia, both resolved within 24 h. No increases in suicidal ideation emerged, and no suicidal behaviors were reported in the post-dosing follow-up visits. No serious adverse events were detected; see Table 1 for details).

The secondary outcomes were changes in eating psychopathology, BMI, and general psychopathology assessed up to 3 months after treatment. Weight concerns significantly decreased from baseline to 1-month and 3-month follow-up, with a medium to large effect. Shape concerns significantly decreased at the 1-month follow-up but not at the 3-month follow-up. Eating concerns significantly decreased only at the 3-month follow-up. Dietary restraints did not display any significant changes. Interestingly, 4 participants out of 10 exhibited a decrease in global EDE-Q scores to within 1 standard deviation (SD) of community norms. Mean BMI did not show any statistically significant changes, but 5 participants evidenced an increase at the 3-month follow-up (range 0.4–1.2 kg/m^2^). At the 1-month follow-up, significant reductions were reported concerning the trait body image anxiety, trait anxiety, functional impairment related to disordered eating, and preoccupations and rituals surrounding food, eating and shape. Finally, the psilocybin treatment was well-accepted and considered meaningful by all participants. Nine participants stated that one dosing session was too few. There was no need for anxiolytic rescue medication during the session.

### Registered clinical trials

Six registered clinical trials employing psilocybin in people suffering from EDs have been identified on the website https://clinicaltrials.gov. The characteristics are detailed in Table 2.

One (NCT05035927) is an open-label study on people suffering from BED, based on Gainesville, FL. The protocol was submitted in 2021, the recruitment started in 2022, and the completion is estimated in August 2025. The status is currently “active, not recruiting”. The treatment consists of a single 25 mg dose of psilocybin 6–8 h after preparatory psychotherapy. The study aims to involve 10 participants and assess the safety, feasibility, magnitude and duration of dissociative effects, frequency of binge eating episodes, waist circumference, and weight. Researchers will evaluate the outcomes through adverse effects monitoring, laboratory tests, vital signs, the Mystical Experience Questionnaire (MEQ30), the Magnetic Resonance Spectroscopy (MRS), and the measurement of binge eating episodes and physical parameters, at 6 and 12 weeks after treatment. The results have not been shared yet.

The other 5 protocols are focused on people suffering from AN (1 specifying AN-R subtype). For 3 of them the status is “completed”, 1 is “recruiting”, and 1 is “not yet recruiting”. Four are open-label studies, and 1 is an RCT.

Only one registered clinical trial (NCT04661514) published the results, which is the open-label study we included in our systematic review, and that we previously described [63].

The only RCT (NCT05481736) is a multicentric (San Diego, CA; Baltimore, MD; New York, NY; Austin, TX; Dublin, IRE; London, UK) phase 2 study that started recruitment in October 2022 and estimated completion in June 2024. The status is currently “recruiting”, and the expected sample size is 60 participants. The study design is a parallel assignment with randomized allocation and quadruple masking (participants, care providers, investigators, and outcomes assessors). The experimental treatment is a dose of 25 mg of psilocybin compared with 1 mg of psilocybin. The study aims to assess safety, and improvement in eating psychopathology, weight, and general psychopathology. Researchers will evaluate the outcomes through adverse events monitoring, the Eating Disorder Examination (EDE), the Yale-Brown Obsessive Compulsive Scale (Y-BOCS), and BMI, at 4-week and 12-week follow-ups.

The remaining 3 registered clinical trials (NCT04052568, NCT04505189, NCT06399263) are open-label studies. One is based on Baltimore, MD, one in London, UK, and one in San Francisco, CA. The status of 2 of them is “completed” but not published the results yet, 1 is “not yet recruiting” with estimated completion in June 2029. The sample size ranges from 21 to 40 participants. The treatment of one study consisted of up to 4 sessions with a dose of 20–30 mg of psilocybin; another study 3 sessions of 25 mg of psilocybin; the last study 2 sessions with 20 mg and 30 mg of psilocybin. One study aimed to explore changes in eating and general psychopathology, weight, and health-related quality of life through the assessment of the Hospital Anxiety and Depression Scale (HADS), the Eating Disorder Quality of Life Scale (EDQLS), the EDE-Q, the EDE, BMI, and the Anorexia Nervosa Stages of Change Questionnaire (ANSOCQ), at 1-week, 1-month, 2-month, and 3-month follow-ups. One study assessed changes after 6 months in Functional Magnetic Resonance Imaging (fMRI), and in eating psychopathology with the EDE, the EDE-Q, and the Readiness and Motivation Questionnaire (RMQ). The last assesses changes in eating psychopathology with the EDE at baseline, 28 days, 90 days, and 365 days after treatment.

### Quality assessment

Based on the characteristics of the studies, we applied the *Quality Assessment Tool for Before–After (Pre–Post) Studies With No Control Group* released in 2013 by the National Heart, Lung, and Blood Institute [64]. Single-item results are detailed in supplementary material Table S1. One study [63] had a global quality score of 15/24, and the other [61] 4/24.

## Discussion

Notwithstanding the limits due to the small sample size, the first findings evidence that psilocybin is safe, feasible, and well-tolerated in EDs. In addition, preclinic data [30, 32, 33, 65] and studies on healthy volunteers [32, 33, 66–68] suggest a potential benefit in the treatment of EDs. These early results support the need for further research.

The open-label feasibility study [63] showed that 25 mg of psilocybin in conjunction with psychological support was safe and well-tolerated by 10 people with AN. Two cases of hypoglycemia occurred, resolving in less than 24 h without intervention. This effect might be because psilocybin increases the brain and body glucose consumption, possibly via the interaction with cerebral receptors and cortisol, thus reducing the blood levels in a population with scarce energy reserves and predisposed to hypoglycemia [69–71]. This event indicates the importance of monitoring blood glucose before, during, and after treatment, and being prepared to supply glucose if needed. No participants reported serious adverse effects, and no other laboratory changes were evidenced. In this study, weight concerns improved both after 1 and 3 months, eating concerns after 3 months, and shape concerns after 1 month. Dietary restraints did not change significantly. These improvements could be explained as psilocybin can reduce the activity of areas responsible for cognitive rigidity and ruminations (e.g., *DMN* and CCA) while increasing the insula activity [30, 33–37]. This evidence of body weight maintenance and cognitive flexibility improvement was also confirmed by studies in which psilocybin was administered to ABA rodent models [38]. Dietary restraints might instead require a longer time or more complex treatments to regress. BMI did not display any significant changes at the follow-up. This is not surprising, since BMI requires prolonged periods of treatment and eating psychopathology improvements to increase [72, 73]. Four 4 participants out of 10 demonstrated significant improvements in the global EDE scores at the 3-month follow-up, with values reaching 1 SD of community normative values. Of those participants, 2 showed positive BMI trends, 1 showed a stable trend in normal ranges, and only 1 a decrease. This indicates that psilocybin might be a treatment whose efficacy is variable among people suffering from AN. Further studies with enlarged samples are required to identify which types of patient psilocybin is more beneficial. The treatment was also favorable regarding trait body image anxiety, trait anxiety, and preoccupations and rituals surrounding food, eating, and shape. These results confirm that psilocybin might be useful in AN to manage comorbidities and general psychopathology [22–24, 74].

Finally, none of the 10 participants dropped out of the 3-month follow-up, quality of life overall improved, and most reported the experience as highly meaningful and with a positive life impact. This finding is notable, since drop-outs in EDs can reach up to 40%, and this directly affects clinical outcomes [75–77]. The positive engagement could also reduce resistance to treatment, which is beneficial for people suffering from AN [78]. Nine participants claimed that one single session was not sufficient. Researchers planning future studies should take this into account, considering that persons suffering from AN can handle two or more doses of psilocybin.

The case report showed no adverse effects, and the participant well-tolerated the two injections of psilocybin [61]. After 1 month, weight increased significantly, from 40 to 47 kg (BMI from 14.0 to 16.4).

The authors described the patient as a perfectionist, obsessive and self-centered, with no emotional or sexual life and marked religiousness. Perfectionism and obsessionality are closely associated with AN [79–82]. Since psilocybin reduces the activity of areas linked to cognitive rigidity, individuals with these traits may particularly benefit from this treatment. Further studies with larger cohorts are required to confirm or deny this assumption.

Despite treatment with chlorpromazine, the patient exhibited depressive-like symptoms, including asthenia, insomnia, dark thoughts, and lack of confidence. Mood improvement rapidly, in parallel with weight gain. This finding is congruent with previous research in which psilocybin showed promising results in treatment-resistant depression [23, 83]. Mood improvement may have partially driven the weight recovery, given the influence of depressive symptoms on eating psychopathology [49].

The authors provided a rich description of the contents and emotions the patient felt after psilocybin injections. Among the emotions expressed, feelings of guilt were reported. These emotions have a negative correlation with binge-purging symptoms in AN [84]. Working through these feelings after treatment might have helped resolve inner conflicts that contributed to perpetuating eating symptomatology. Aligns with the RElaxed Beliefs Under pSychedelics (REBUS) model, which posits that psychedelics loosen rigid prior beliefs, enabling new insights and changes in body self-perception [42, 43]. The “pivotal mental states” model further asserts that psychedelic treatments promote a hyper-plastic state that allows deep learning and psychological transformation [85]. Treatments based on these models may also help process and overcome traumatic experiences, which might foster the onset and perpetuation of EDs [33, 86, 87].

Finally, it is notable that the patient was extremely underweight (BMI: 14.0) and had a long course of the disease (16 years of illness). That the treatment was well-tolerated and efficient in one case of SE-AN is encouraging. In fact, among these persons, effective treatments are lacking, and mortality reaches up to 20% [7, 12, 88]. Thus, psilocybin appears to be a potentially safe and promising option for a condition still lacking effective therapies.

Six clinical trials employing psilocybin in EDs have been registered, 5 for AN and 1 for BED. Five are open-label studies with a single-group assignment design, and one is an RCT comparing two different doses of psilocybin. Only one has published the results, which is the open-label study we included. Two trials have been completed, and the findings have not yet been shared, so new data will likely be available in the near future. The novelty of the treatment is underlined by the fact that five trials out of six were registered after 2020. The hope is that investments and research in this molecule will increase in future years. Given the safety and promising results obtained in preliminary studies, and the paucity of trials currently registered, we might assert that large space is available and there is a need for new studies on psilocybin in EDs. Funding for these studies should be increased, as also experts claim [51, 53].

## Strengths and limits

This review provides a comprehensive overview of the current state of research on psilocybin as a potential treatment for EDs. This is the first systematic review to synthesize findings on psilocybin in EDs, highlighting its safety profile and therapeutic benefits. They were evaluated both published studies and registered clinical trials, offering the broadest perspective on the available evidence.

However, some limitations must be acknowledged. First, evidence is scarce at present. Only one open-label feasibility study and a case report have been published. In addition, the number of participants was narrow, and the outcomes mostly consisted of self-report measures. Finally, given the novelty of this research area, long-term effects remain largely unexplored. Future studies should focus on larger, well-controlled trials to establish efficacy and safety more robustly.

## What is already known on this subject?

New treatments in EDs are required, since a large part of the population could not reach recovery. Psilocybin has been increasingly investigated as a potential treatment for various psychiatric conditions, including major depressive disorder, treatment-resistant depression, substance use disorders, and obsessive–compulsive disorder. The rationale for utilizing psilocybin in EDs is solid, both on a biological and psychological level. The safety profile of psilocybin is considerably favorable in healthy subjects. However, it is reasonable to be concerned that the effects of psilocybin in individuals with EDs may differ.

## What your study adds?

This systematic review is the first to provide a comprehensive synthesis of the existing literature on psilocybin in EDs. Preliminary findings suggest that psilocybin might be well-tolerated in persons suffering from AN. In addition, the first exploratory data indicate potential benefits in reducing eating psychopathology, even in a case of SE-AN. Further research is required to validate these early findings, particularly clinical trials with larger samples. Psilocybin in EDs is currently being explored in 6 clinical trials, so new data are expected in the following years.

## Supplementary Information

Below is the link to the electronic supplementary material.

Supplementary file1 (DOCX 19 KB) Quality assessment of included studies assessed through the *Quality Assessment Tool for Before–After (Pre–Post) Studies With No Control Group* released by the National Heart, Lung, and Blood Institute (NHLBI)

## Data Availability

No data sets were generated or analyzed during the current study.
